# P-132. Retrospective Comparison of Short and Prolonged Treatment Strategies for Patients with Neutropenic Enterocolitis

**DOI:** 10.1093/ofid/ofae631.337

**Published:** 2025-01-29

**Authors:** Tori Pravato, Arsheena Yassin, Navaneeth Narayanan, Sana M Mohayya, Ahmed Abdul Azim, David A Awad, Junhee Cho, Lianna Schwartz-Orbach, Pinki Bhatt

**Affiliations:** Robert Wood Johnson University Hospital - New Brunswick, Staten Island, New York; Robert Wood Johnson University Hospital, New Brunswick, New Jersey; Rutgers University Ernest Mario School of Pharmacy & Robert Wood Johnson University Hospital, New Brunswick, NJ; Robert Wood Johnson Barnabas Health, New Brunswick, New Jersey; Rutgers Robert Wood Johnson Medical School, New Brunswick, New Jersey; Robert Wood Johnson University Hospital, New Brunswick, New Jersey; Ernest Mario School of Pharmacy, New Brunswick, New Jersey; Robert Wood Johnson Medical School of Rutgers University, Hartford, Connecticut; Rutgers - Robert Wood Johnson Medical School, New Brunswick, New Jersey

## Abstract

**Background:**

Neutropenic enterocolitis (NE) is a severe necrotizing condition affecting the small and/or large intestines in neutropenic patients. With a lack of high-quality studies looking at antimicrobial duration and outcomes for patients with NE, this study aims to evaluate the impact of short versus prolonged antimicrobial treatment durations on clinical outcomes in patients with NE.

**Figure:**
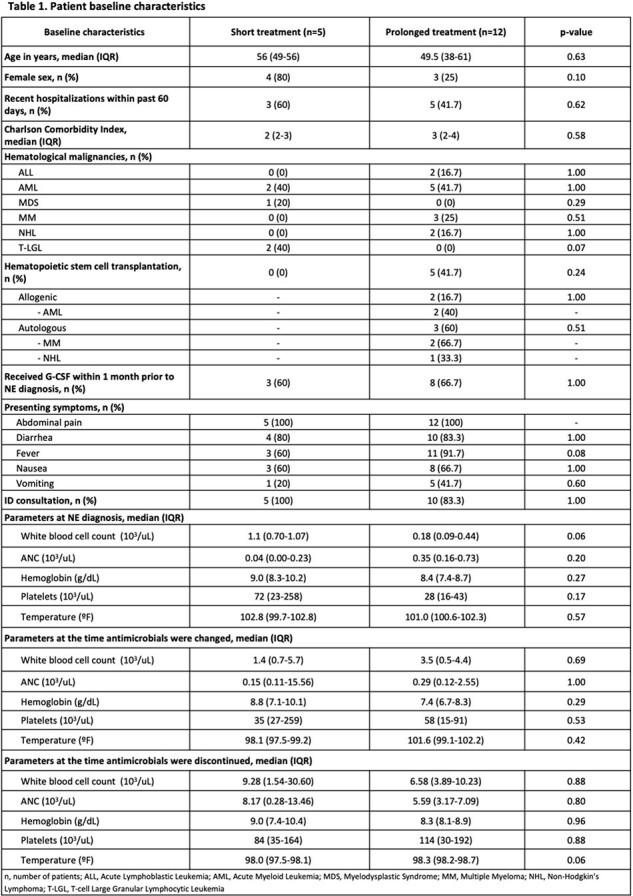

**Methods:**

This was a single-center, retrospective, cohort study conducted at a tertiary care academic medical center in New Jersey. Medical records of adult patients with hematologic malignancies admitted between January 2015 to September 2023, with diagnosis of NE and receipt of treatment with broad-spectrum antimicrobials, were reviewed. This study compared patients who received short versus prolonged antimicrobial treatment courses for NE. Patients in the short treatment group included those who received broad antimicrobials for NE treatment, that were stopped or switched to prophylaxis while the patient’s absolute neutrophil count (ANC) was less than 0.5 x 10^3^/uL. The prolonged treatment group included patients who received broad antimicrobials at least until ANC was over 0.5 x 10^3^/uL. The primary endpoint was the time to clinical improvement of symptoms between the two groups. Secondary endpoints included hospital length of stay (LOS), intensive care unit (ICU) admission, incidence of NE complications, and 30-day mortality.

**Figure:**
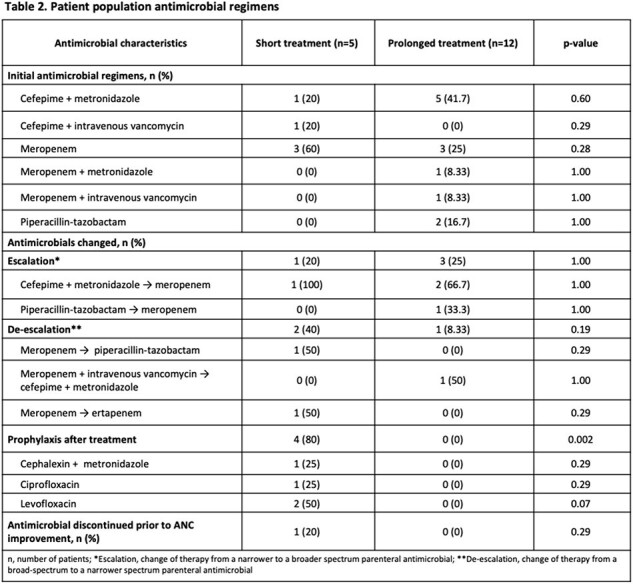

**Results:**

A total of 17 patients were included, with 5 (21.7%) patients who underwent hematopoietic stem cell transplantation. Time to clinical improvement, defined as improvement in symptoms (including resolution of abdominal pain, abdominal distension and/or cramps, diarrhea, nausea, vomiting, lower GI bleed), and 30-day mortality were similar in both treatment groups. However, hospital LOS, ICU admission, and incidence of NE complications were higher in the prolonged treatment group.

**Figure:**
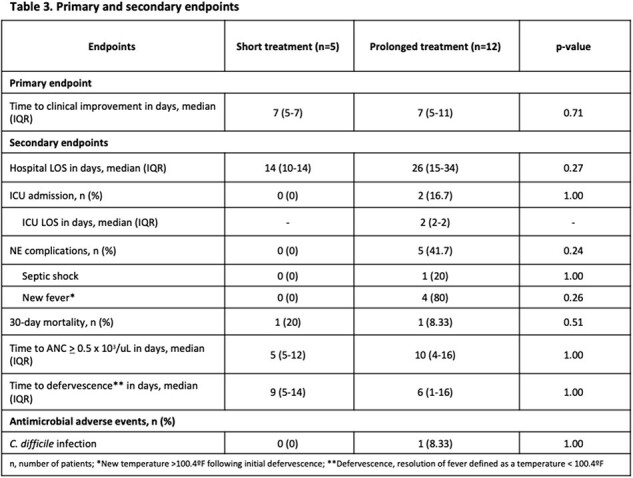

**Conclusion:**

Between short and prolonged treatment groups in patients with NE, time to clinical improvement was similar. Larger, randomized controlled trials are needed in this patient population to assess the appropriate duration of therapy for patients with NE.

**Disclosures:**

**Navaneeth Narayanan, PharmD, MPH, BCIDP**, Astellas: Honoraria|Beckman Coulter: Honoraria|Merck: Grant/Research Support|Shionogi: Grant/Research Support **Pinki Bhatt, MD**, Sanofi: Grant/Research Support

